# Competitive Growth Assay of Mutagenized *Chlamydomonas reinhardtii* Compatible With the International Space Station Veggie Plant Growth Chamber

**DOI:** 10.3389/fpls.2020.00631

**Published:** 2020-05-25

**Authors:** Junya Zhang, Bárbara S. F. Müller, Kevin N. Tyre, Hope L. Hersh, Fang Bai, Ying Hu, Marcio F. R. Resende, Bala Rathinasabapathi, A. Mark Settles

**Affiliations:** ^1^Horticultural Sciences Department, University of Florida, Gainesville, FL, United States; ^2^Center for the Advancement of Science in Space, Melbourne, FL, United States

**Keywords:** algae, space environment, genetic fitness, population genetics, *Chlamydomonas reinhardtii*

## Abstract

A biological life support system for spaceflight would capture carbon dioxide waste produced by living and working in space to generate useful organic compounds. Photosynthesis is the primary mechanism to fix carbon into organic molecules. Microalgae are highly efficient at converting light, water, and carbon dioxide into biomass, particularly under limiting, artificial light conditions that are a necessity in space photosynthetic production. Although there is great promise in developing algae for chemical or food production in space, most spaceflight algae growth studies have been conducted on solid agar-media to avoid handling liquids in microgravity. Here we report that breathable plastic tissue culture bags can support robust growth of *Chlamydomonas reinhardtii* in the Veggie plant growth chamber, which is used on the International Space Station (ISS) to grow terrestrial plants. Live cultures can be stored for at least 1 month in the bags at room temperature. The gene set required for growth in these photobioreactors was tested using a competitive growth assay with mutations induced by short-wave ultraviolet light (UVC) mutagenesis in either wild-type (CC-5082) or *cw15* mutant (CC-1883) strains at the start of the assay. Genome sequencing identified UVC-induced mutations, which were enriched for transversions and non-synonymous mutations relative to natural variants among laboratory strains. Genes with mutations indicating positive selection were enriched for information processing genes related to DNA repair, RNA processing, translation, cytoskeletal motors, kinases, and ABC transporters. These data suggest that modification of DNA repair, signal transduction, and metabolite transport may be needed to improve growth rates in this spaceflight production system.

## Introduction

Microalgae grow by converting light, water, and CO_2_ into biomass. Algae have long been proposed for space life support systems to recycle CO_2_ and provide food either directly or indirectly to astronauts (Brechignac and Schiller, 1992; Ai et al., 2008; Niederwieser et al., 2018; Matula and Nabity, 2019). Many species of microalgae are photosynthetically efficient under the limiting light and low volume conditions necessary in space production (Kliphuis et al., 2012). As single cell organisms, microalgae are easy to cultivate with minimal requirements, and have great potential to yield value-added products.

Both eukaryotic and prokaryotic species, such as *Chlorella vulgaris* and *Arthrospira platensis*, respectively, are generally regarded as safe (GRAS) for human consumption (Caporgno and Mathys, 2018). Algae have nutritional benefits with high levels of antioxidants and protein with essential amino acids (Buono et al., 2014). Algae are also rich in ω-3 fatty acids, such as eicosapentaenoic acid and docosahexaenoic acid (Salem and Eggersdorfer, 2015). The red algal carotenoid, astaxanthin, has multiple uses including the ability to protect against retinal damage in animals (Wang et al., 2000; Katagiri et al., 2012; Liu et al., 2016; Otsuka et al., 2016; Shah et al., 2016). Algal oils may be used in future astronaut diets to help mitigate harmful effects of microgravity and cosmic radiation during spaceflight. *Chlamydomonas reinhardtii* is a model organism for unicellular algae with a well-annotated genome sequence (Merchant et al., 2007). Although Chlamydomonas is not designated as a GRAS organism, feeding studies show no harmful effects of including significant amounts of algae biomass in animal feed (Baek et al., 2018; Murbach et al., 2018). Consequently, Chlamydomonas can be used as a model for other microalgae species, an animal feed for space food systems, or a feedstock for manufacturing.

A few past studies exposed or grew algal species in space conditions (Mergenhagen and Mergenhagen, 1989; Wang et al., 2006; Giardi et al., 2013; Niederwieser et al., 2018). A Space Shuttle mission in 1985 used *C. reinhardtii* to examine phototaxic responses in microgravity (Mergenhagen and Mergenhagen, 1989). Microgravity allowed cells to remain close to light sources in spaceflight suggesting that photosynthetic productivity could be higher in space. By contrast, Wang et al. (2006) grew cyanobacteria for 15 days in a satellite and found reduced growth relative to a ground control. However, the ground control temperature and light cycles were not adjusted to the space conditions. The satellite had two temperature drops and two missed day photoperiods preventing strong conclusions from being drawn about growth rates. Giardi et al. (2013) compared Chlamydomonas photosynthetic responses for wild-type and photosystem II (PSII) D1 mutants in stationary cultures. Spaceflight had a greater negative effect on PSII fluorescence in wild-type cells and negatively impacted cell growth upon return to Earth. Soviet and Russian spaceflight experiments indicate that *C. vulgaris* has similar growth kinetics in space and on Earth, but the cells experience spaceflight as a stress (reviewed in Niederwieser et al., 2018).

The impact of the spaceflight environment for larger scale production of algae is currently unknown. To test larger scale microalgae production, the European Space Agency (ESA) has developed a photobioreactor that pumps liquid media through a meandering path, pipe-reactor (Bretschneider et al., 2016). Gas exchange is mediated by fluorinated ethylene propylene (FEP) membranes that allow CO_2_ and O_2_ diffusion, while algae are continuously mixed with a peristaltic pump (Helisch et al., 2019). This photobioreactor is currently being tested on the International Space Station (ISS) for long-term growth of *C. vulgaris.* A significant challenge of maintaining photosynthetic productivity is regular removal of stationary cells and addition of new media to maintain photoautotrophic growth without forming excessive biofilms within the raceway. In addition, this photobioreactor experiment is not focusing on understanding genes required for growth in spaceflight conditions (Helisch et al., 2019).

In yeast, competitive growth experiments in liquid culture were used to identify genes needed for survival in spaceflight conditions (Nislow et al., 2015). We are completing spaceflight experiments to determine similar gene sets for Chlamydomonas via competitive growth of mutagenized cells. Here we report results from our development of methods and experiment verification test (EVT). We describe a simple batch culture protocol using commercial FEP tissue culture bags that is adapted to the Veggie plant growth chambers on the ISS. Shortwave ultraviolet (UVC) light mutagenesis and full genome sequencing enabled the detection of new mutations in two microalgae strains. The spectrum of mutations identified suggests that UVC primarily induces DNA damage in Chlamydomonas via errors in translesion synthesis and double strand break repair. In addition, a variety of cellular information processing functions may need to be modified to adapt Chlamydomonas to this batch culture system.

## Materials and Methods

### Strains and Culturing Conditions

Strains CC-5082 (WT) and CC-1883 (*cw15*) were obtained from the Chlamydomonas Resource Center. CC-5082 is a sequence-verified clone of CC-1690, which is a wild-type strain of mating type *mt*^+^ (Gallaher et al., 2015). CC-1883 is an *mt^–^ cw15* cell wall mutant, which allows transformation of exogenous DNA by vortexing with cells and glass beads (Kindle et al., 1989). Strains were maintained at room temperature (20–25°C) on agar plates with Tris acetate-phosphate (TAP) medium (Gorman and Levine, 1965), under 50–100 μmol/m^2^/s continuous photosynthetically active radiation (PAR) from daylight fluorescent bulbs (6500 K).

To initiate liquid cultures, 1–2 mm colonies were scraped from TAP agar plate and inoculated into 50–100 mL TAP media. Traditional liquid cultures were grown with continuous light in 250 mL Erlenmeyer flasks with 100 r/min gyratory shaking. For spaceflight analogs, liquid cultures were grown with continuous light in 120 mL PermaLife cell culture bags (OriGen Biomedical, Austin, TX, United States). Liquid cultures were grown in daylight fluorescent lighting or in the Veggie Vegetable Production System at the Kennedy Space Center (Merritt Island, FL, United States) with a ratio of red (630 nm): green (525 nm): blue (450 nm) of 8:1:1 (Massa et al., 2016). The Veggie reservoir was set at the maximum distance from the LED lightcap. All lighting was 80–100 μmol/m^2^/s.

The UVC mutagenesis dose that caused ∼10% cell survival was determined by transferring 7 mL of early-log phase culture at OD_600_ = 0.45 to 15 cm sterile petri plates in a sterile laminar flow hood. The petri plates were opened in a GS Gene Linker UV Chamber (Bio-Rad, Hercules, CA, United States) and exposed to increasing doses of UVC light from germicidal bulbs. Petri plates were closed, wrapped in aluminum foil, and agitated overnight in the dark at 50 r/min. Mutagenized cultures were plated on TAP agar plates with non-treated samples diluted 1:5 prior to plating. Colonies were grown under continuous light, counted, and normalized relative to non-mutagenized cultures.

### EVT Mutagenesis

Colonies from TAP agar plates were scraped and suspended in 600 μL liquid TAP media and adjusted to an optical density of 1 at 600 nm using a visible light spectrophotometer (SmartSpec 3000, Bio-Rad, Hercules, CA, United States). The Chlamydomonas suspension was used to inoculate TAP liquid media at a 1:250 dilution, i.e., 0.2 mL of OD_600_ = 1 suspension was added to 50 mL TAP media. WT was inoculated on day 1 of the experiment and *cw15* was inoculated on day 2. The cultures were grown in flasks until reaching an OD_600_ of 0.4–0.5 on day 4. Non-mutagenized cells from the culture were sampled for whole genome sequencing by centrifuging 2 mL of the culture and freezing the cell pellet at −80°C until DNA was extracted. For each mutagenesis, 7 mL of early-log phase culture was exposed to 8 mJ of UV light. The mutagenized cells were then used to inoculate 100 mL of TAP media in a PermaLife tissue culture bag in a sterile laminar flow hood. Inoculated tissue culture bags were held at room temperature in the dark for 7 days and then transferred to the Veggie growth chamber.

### EVT Culture Conditions and Selection

The Veggie chamber was set with the reservoir at maximum distance from the lighting (Massa et al., 2016). The 630 nm red light was set to “medium”; the 450 nm blue light was set to “low”; and the 525 nm green light was set to “on.” The bellows were closed during growth cycles, and the fan was set to “low.” The initial mutagenized cultures were grown for 7 days in Veggie. The cultures were then passaged by transferring 1 mL of culture to a bag containing fresh TAP media using a sterile syringe. The second culture was grown for 6 days and then passaged to a third tissue culture bag for 6 days of growth. During each passage, 2 mL of culture was sampled, centrifuged, and the cell pellet was frozen at −80°C until DNA was extracted. The remaining cultures were stored in a soft stowage, Cargo Transport Bag (CTB) until 36 days after the initial inoculation. For each dark-stored culture, 2 mL was sampled for DNA extraction.

### Growth Assays

Growth curves for non-mutagenized cells were determined by sampling 1 mL of culture each day and reading the absorbance at 600 and 750 nm. Culture samples were diluted if optical density measurements were greater than 1. Cell density was counted using a hemocytometer and light microscopy. Dry biomass was determined by transferring all of the remaining culture volume into two 50 mL centrifuge tubes. The cells were centrifuged with the supernatant media removed and the cell pellets were lyophilized overnight. Dry weights were measured on an analytical balance. Biomass for each culture is the average of the two technical replicates.

### Whole Genome Sequencing

DNA was extracted from the flash-frozen and dark-stored cell pellets as described with some minor modifications (Newman et al., 1990). Cell pellets were resuspended in 150 μL H_2_O on ice, and 300 μL of SDS-EB buffer (2% SDS, 400 mM NaCl, 40 mM EDTA, 100 mM Tris-HCl, pH 8.0) was added and vortexed. The cell suspension was then extracted with 350 μL phenol: chloroform: isoamyl alcohol 25:24:1 (v:v) for 2–5 min by intermittent mixing using a vortex. The organic and aqueous phases were separated with a 5 min centrifugation at maximum speed in a microcentrifuge. The aqueous phase was then extracted with 300 μL chloroform: isoamyl alcohol (24:1). The RNA in the aqueous phase was digested with RNase A at room temperature for 10 min. DNA was precipitated by adding 1 mL 100% ethanol, incubating on ice for 30 min, and centrifuging for 10 min. The pellet was washed with 200 μL 70% ethanol, dried in a vacuum centrifuge, and resuspended in 30 μL H_2_O. DNA sample integrity was evaluated with an Agilent TapeStation (Santa Clara, CA, United States), and concentration was determined with Qubit dsDNA HS Assay Kit (Life Technologies, Carlsbad, CA, United States) according to the manufacturer’s instructions.

For each library, 3 μg of DNA was sheared to 350 bp average size using an S220 Focused-ultrasonicator (Covaris, Woburn, MA, United States). Barcoded TruSeq PCR-Free Low Throughput libraries were prepared for each DNA sample following the manufacturer’s instructions (Illumina, San Diego, CA, United States). Each sample had a unique index from Illumina TruSeq DNA CD Indexes (96 indexes/samples). The libraries were split into two pools to sequence a total of 38 samples: nine dark-stored and nine flash-frozen samples for each strain, growth cycle, and biological replicate as well as the two non-mutagenized initial cultures. Library pools were color-balanced and sequenced with paired-end 150 bp reads on the Illumina HiSeq X platform at MedGenome (Foster City, CA, United States).

### Read Processing and Genome Alignment

The raw sequence reads were filtered using Trimmomatic v.0.38 to remove barcode and adaptor sequences in paired-end mode (Bolger et al., 2014). High quality reads were aligned to the *C. reinhardtii* reference genome (Merchant et al., 2007), v.5.6 from Phytozome, using BWA-mem (Li, 2013). Duplicate reads were removed using Picard MarkDuplicates, v.2.19.1^1^ with default parameters. Reads near insertion-deletion (InDel) polymorphisms were realigned using GATK v.3.8.1 RealignerTargetCreator and IndelRealigner (McKenna et al., 2010; DePristo et al., 2011).

### Identifying Sequence Variants

Three software packages for detecting single nucleotide polymorphisms (SNPs) and small indels were compared. FreeBayes v.1.2.0 (Garrison and Marth, 2012) was implemented with the pooled continuous option. LoFreq v. 2.1.3.1 used default parameters (Wilm et al., 2012), while CRISP pooled, discrete parameters were: –poolsize 5 –perms 100000 –mmq 10 –minc 3 (Bansal, 2010; Bansal et al., 2011). FreeBayes called more variants for libraries with the lowest depth giving a negative correlation between the number of mapped reads and number of variants detected. LoFreq is restricted to calling variants for single samples and not detecting variants at a population level. Consequently, CRISP was determined to be the optimal approach for variant detection.

For each strain, CRISP genotyping results were filtered to remove variants with a call rate below 70% (i.e., missing data ≥ 30%) and monomorphic variants with a minor allele frequency (MAF) of zero. Monomorphic variants revealed non-mutated, natural variants relative to the reference genome. In addition, variants with low depth and low-quality mapped reads were removed based on CRISP assignments of “LowDepth, LowMQ10 and LowMQ20.” Finally, novel variants were scored by identifying exact matches to natural variants reported from whole genome sequencing of 39 Chlamydomonas laboratory strains (Gallaher et al., 2015). Variant density was visualized by plotting SNPs detected in windows of 100, 400, 500, and 1000 kb. A window size of 400 kb was selected visually as showing the highest resolution of natural variant SNP clusters.

### Determining Protein Coding Variants and Selection

The effect of each variant on protein coding regions was predicted using SnpEff v.4.3t (Cingolani et al., 2012). Synonymous and non-synonymous mutations from the primary SnpEff call were used to estimate the overall impact of UVC mutagenesis on protein coding genes. Synonymous (π_S_) and non-synonymous (π_N_) nucleotide diversity was estimated for each gene using SNPGenie (Nelson et al., 2015). SNPGenie was run for all high-quality and novel variants independently, using both the forward strand and reverse complement strand. Positive selection was inferred when π_N_ > π_S_, and purifying selection was inferred when π_N_ < π_S_. Genes showing positive or purifying selection were tested for gene ontology (GO) term enrichment using AgriGO v2.0 with default parameters except for the “Hochberg (FDR)” multi-test adjustment method for the default false discovery rate of 0.05 (Tian et al., 2017).

## Results

**FIGURE 1:**
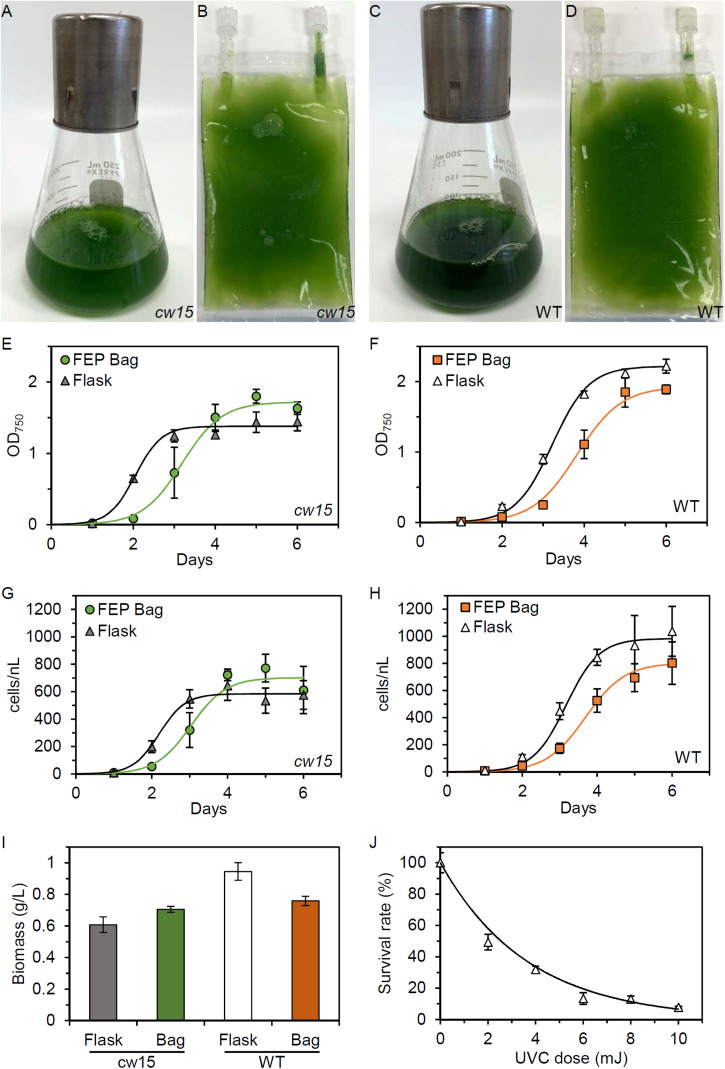
Microalgae growth in FEP plastic culture bags. **(A)** Flask and **(B)** FEP bag culture of *cw15*, CC-1883. **(C)** Flask and **(D)** FEP bag culture of wild-type (WT), CC-5082. Growth curves of *cw15* **(E,G)** and WT **(F,H)** algae comparing flask and FEP bag cultures. Symbols are mean values (*n* = 5, except *n* = 4 for *cw15* FEP bags). Trend lines are logistic growth curve regressions. **(I)** Average biomass produced after 6 days of culture. **(J)** Dose-response of WT algae to UVC light. Trend line is an exponential regression. Error bars in all panels are standard deviation.

We tested commercial FEP tissue culture bags for the ability to support microalgae growth without agitation or active mixing of gases with liquids. Under daylight fluorescent lighting, the bags are able to support robust growth for both *cw15* and WT strains (Figures 1A–D). Time courses of growth show a 1 day delay in the bags (Figures 1E–H). Logistic growth curve regressions of optical density estimated the maximum doubling time for *cw15* was 5.32 h in flasks and 8.01 h in FEP bags (Figure 1E). For WT, maximal doubling time was 7.42 h for flasks and 8.98 h for FEP bags (Figure 1F). Similar doubling times were estimated from cell counts: 5.7 versus 7.65 h for *cw15* and 7.3 versus 8.73 h for WT (Figures 1G,H). Cell counts estimated a 600- to 1450-fold increase in cell number during the culture indicating 9–11 cell doublings in the batch cultures. Dry weight biomass was in rank order with the measurements of optical density and cell density at the 6 days time point. The *cw15* flask cultures had the lowest biomass at 0.61 g/L, and the WT flask cultures had the highest biomass at 0.95 g/L (Figure 1I). The FEP tissue culture bags grew to a cell density in-between flask cultures for *cw15* and WT. These data indicate the bags provide sufficient gas exchange to support microalgae, but that Chlamydomonas laboratory strains grow faster in flasks.

To identify genes required for growth in tissue culture bags, we mutagenized the strains prior to competitive growth assays. We first determined the dose-response for cell lethality in a UVC light chamber. Figure 1J shows that 6–10 mJ of UVC exposure is sufficient to kill ∼90% of WT cells. Similar results were obtained for *cw15*, and we concluded that 8 mJ of UVC would give sufficient DNA damage to induce mutations in both strains without risking excessive cell death and culture failure during spaceflight.

**FIGURE 2:**
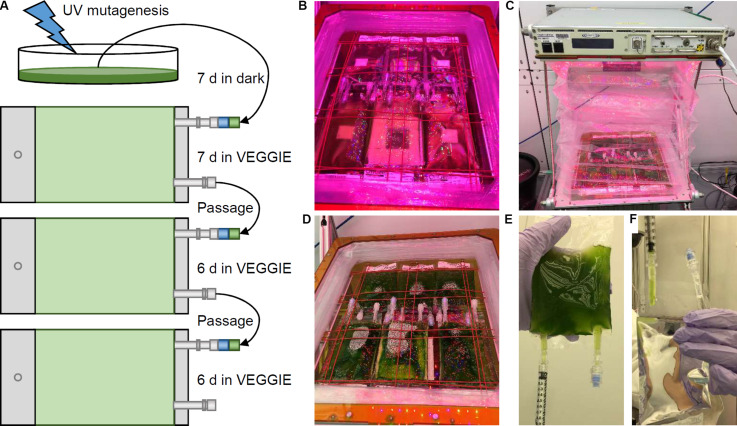
Algae selection experiment in Kennedy Space Center Veggie growth chamber. **(A)** Schematic of the experiment design and workflow. **(B)** Initial installation of mutagenized culture bags in the Veggie reservoir. **(C)** Veggie chamber with algae bags and closed bellows. **(D)** Algae cultures prior to passage. **(E,F)** Passage of culture using sterile syringes.

An EVT was completed at the Kennedy Space Center (Figure 2). Three biological replicates of WT and *cw15* were mutagenized at the University of Florida, Gainesville, FL, United States. The mutagenized cells were transferred to tissue culture bags with TAP media and stored at room temperature in the dark for 7 days to emulate a late load and storage during a resupply mission to the ISS. The cultures were then transferred to a Veggie unit to provide light and stimulate photosynthesis. The culture bags were placed under bungee cords that hold plant pillows in the Veggie reservoir (Figure 2B). The bags were left without any agitation except during passages (Figure 2C). To complete a passage, culture bags were removed from the reservoir, agitated manually, and 1 mL of culture was transferred to fresh media for a new growth cycle (Figures 2D–F). An additional 2 mL of culture was sampled and cell pellets were frozen to preserve a DNA sample without dark storage.

**FIGURE 3:**
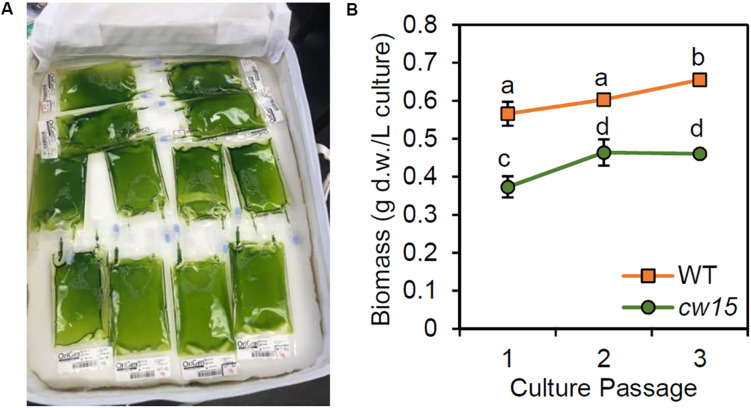
**(A)** Storage of live algae cultures in CTB soft stowage bag. **(B)** Biomass yield after dark storage. Average and standard deviation of three biological replicates are plotted. Lowercase letters indicate statistically significant groups of culture passages based on ANOVA of each strain and a Tukey HSD test.

The remaining culture was stored in a closed CTB to simulate ambient storage on the ISS and return of live cultures to Earth (Figure 3A). At 36 days after the initial inoculation, all culture bags were sampled for DNA extraction and biomass assessment (Figure 3B). The WT strain showed higher biomass compared to the *cw15* cell wall mutant (*p* = 0.01, paired Student’s *t*-test). Within each strain, there was a significant trend for increased biomass with additional culture passages (*p* < 0.01, ANOVA). However, all biomass levels were lower than immediate harvest of cells after 6 days of culture without UV mutagenesis or dark storage (Figure 1I). The biomass trends observed most likely reflect loss of biomass due to respiration during dark storage.

**FIGURE 4:**
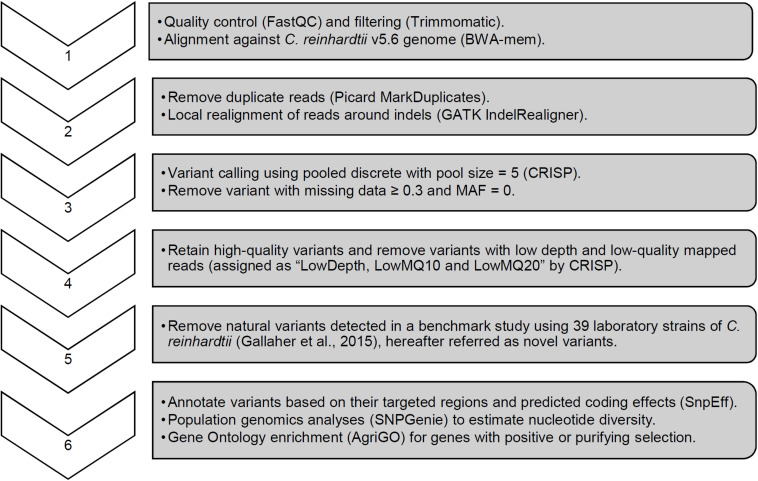
Sequence analysis pipeline with key parameters for variant calling and quality filtering.

Whole genome sequencing of the pre-mutagenized cultures, frozen cell pellets, and dark stored cultures was completed with an average read depth of 16x. Variants consisting of SNPs and short InDel polymorphisms were called with CRISP using pooled sample parameters (Figure 4). After removing missing data (≥30%) and monomorphic polymorphisms (MAF = 0), we detected 73,573 WT variants and 79,455 *cw15* variants. Filtering to remove low-depth and low-quality reads reduced WT and *cw15* polymorphic variants to 48,380 and 53,939, respectively. This represents an average of one variant per 2.24 kbp in WT and 2.01 kbp in *cw15*.

**FIGURE 5:**
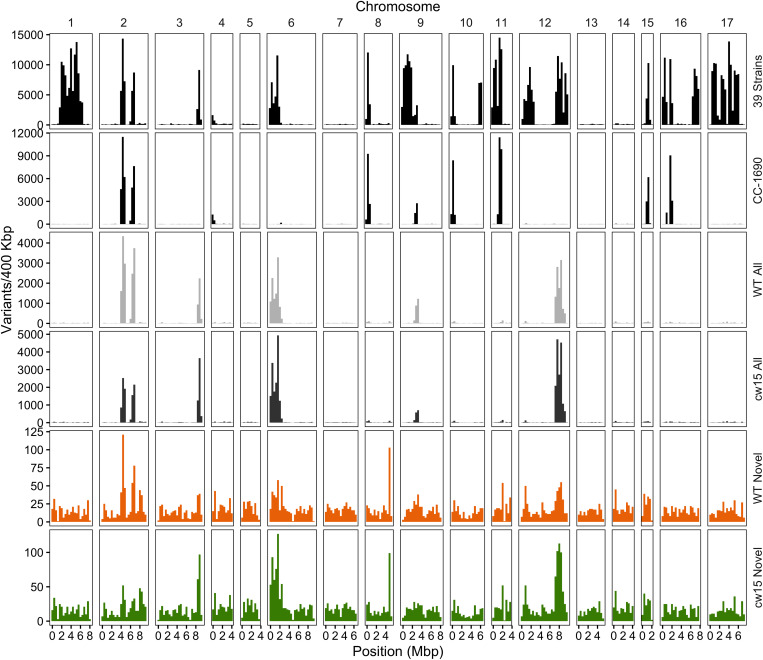
Density of SNP and indel variants in the Chlamydomonas genome. The *Y*-axis is the number of variants per 400 kb, and the *X*-axis is the physical distance of each chromosome. The variant dataset plotted is labeled on the right of each panel. Variants from 39 laboratory strains (black, top panel) and CC-1690 (black, second panel) sequenced by Gallaher et al. (2015). Light and dark gray show all high quality variants (step 4 in Figure 4) in the WT and *cw15* strains. Orange (WT) and green (*cw15*) plot novel variants after filtering identical matches to polymorphisms in the Gallaher et al. (2015) study (step 5 in Figure 4).

Plotting the variant density across the genome identified known hotspots of natural variation among laboratory strains (Figure 5, Gallaher et al., 2015). The WT strain is a sequence-verified clone of CC-1690, and the polymorphic variants identified overlapping peaks on chromosomes 2, 6, and 9. In addition, there were peaks that overlapped with other natural variants on chromosomes 3 and 12. The *cw15* strain derives from a cross with CC-1690 and a similar pattern of natural variant peaks was observed. These natural variants may represent spontaneous mutations that are easily tolerated in Chlamydomonas or regions of the genome that are difficult to align with high confidence. In either case, known natural variants are likely to have little signal of selection due to induced UVC mutagenesis. We removed all exact matches to natural variants to obtain 5286 WT and 5873 *cw15* novel variants, which are more evenly distributed across the genome (Figure 5). The average genomic density for the novel variants were one variant per 20.83 kbp in WT and 18.75 kbp in *cw15*.

**FIGURE 6:**
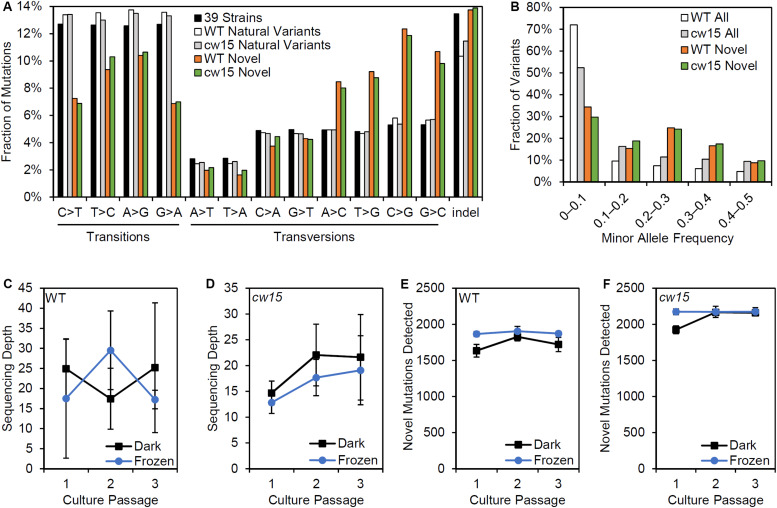
Effects of variant filtering and live culture storage on mutations detected. **(A)** Relative frequency of base changes and indels for natural variants found by Gallaher et al. (2015) and the novel variants after UV mutagenesis. **(B)** Minor allele frequency distribution for all variants and novel variants. **(C,D)** Average sequencing depth of three biological replicate libraries prepared from dark stored cultures and algae pellets that were frozen at the time of passage. **(E,F)** Average number of novel mutations detected in libraries from dark stored and frozen tissues. Error bars are standard deviations.

The novel variants show a different spectrum of base changes than natural variants (Figure 6A). There is a relative decrease in transitions and an increase in transversions with the complementary mutations of A > C and T > G as well as C > G and G > C predominating. These data suggest the novel variants represent mutations caused by induced mutagenesis instead of the endogenous spectrum of Chlamydomonas variants. In addition, the novel mutations are found at a higher allele frequency indicating that the novel mutations have more sequence read support across samples than natural variants (Figure 6B). We conclude that novel variants better represent the UVC mutagenized sites.

Centrifugation and freezing cell pellets for DNA sampling requires more extensive astronaut time and limiting resources on the ISS. We compared the mutations recovered from samples that had been frozen at the time of passage and those from live cultures that had been stored in the dark. There were no significant differences in sequencing depth based on the storage conditions (Figures 6C,D). However, dark storage decreased the number of mutations recovered in passage 1, which were cultures stored for 22 days prior to sampling (Figures 6E,F). Student’s *t*-tests showed a significant reduction of mutations detected for the WT strain (*p* = 0.003), but the reduction was non-significant for *cw15* (*p* = 0.07). For frozen stored libraries, the number of novel mutations detected in each passage was nearly constant indicating sequencing depth was limiting for mutant detection. These results suggest that changes in allele frequency over culture passages are not a reliable indicator of selection for this experiment.

**FIGURE 7:**
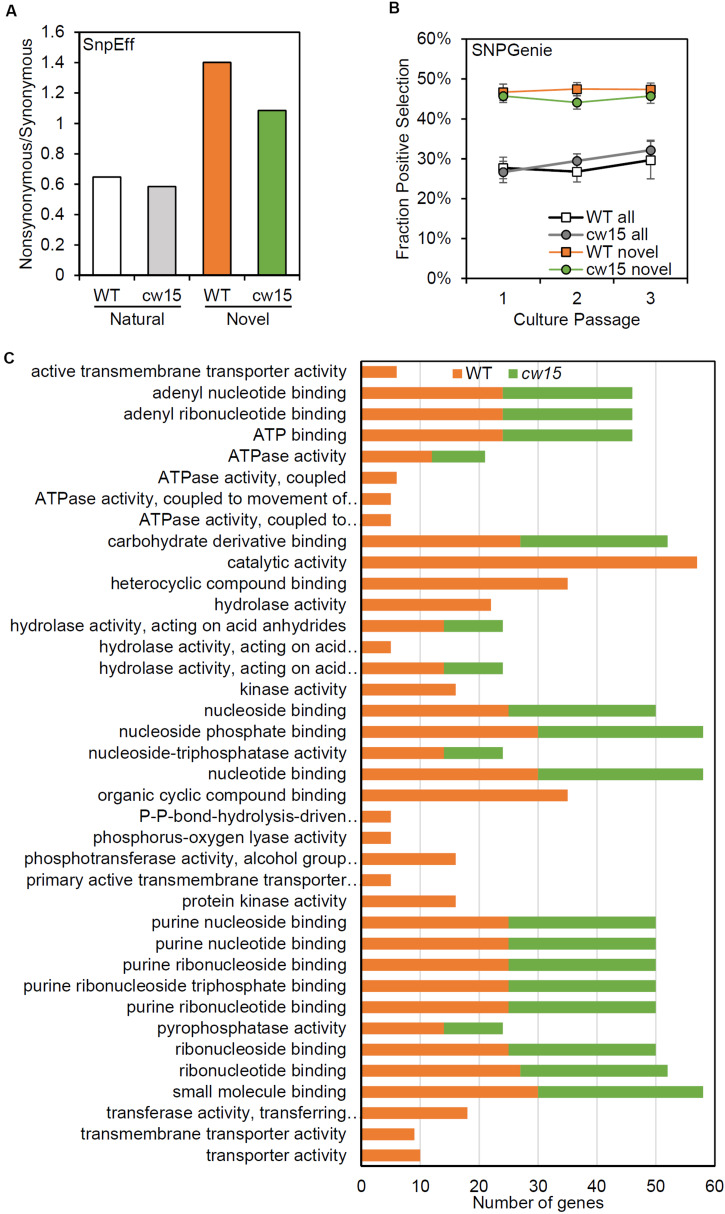
Novel variants are enriched for predicted protein coding changes. **(A)** Ratio of non-synonymous to synonymous variants based on SnpEff annotations. White and gray show natural variants detected. Orange and green show novel variants. **(B)** Average fraction of genes showing positive selection of all genes tested with SNPGenie. Averages are from six libraries per passage. Error bars are standard deviations. **(C)** Enriched GO terms for genes with positive selection.

To assess the effects of novel mutation on protein coding sequences, SnpEff was used to identify protein coding changes. Natural variants were enriched for synonymous mutations, while the novel variants were enriched for protein coding changes (Figure 7A). These results are consistent with an increased frequency of deleterious mutations after UV mutagenesis. Individual genes were tested for selection based on nucleotide diversity (π) using SNPGenie. Novel variants were enriched for genes showing evidence of positive selection with about 46% of genes tested having π_N_ > π_S_, compared to 29% of all variants (Figure 7B). These results are consistent with the enrichment for non-synonymous mutations resulting from UV mutagenesis.

Based on GO term enrichment analyses, the positively selected genes in WT and *cw15* both show significant enrichments for terms associated with purine nucleotide binding and hydrolase activity (Figure 7C). The individual genes with these GO terms represent information processing functions in DNA damage repair, RNA processing, translation, cytoskeletal motors, and signal transduction (Supplementary Table S1). The WT libraries also had enrichment for terms associated with small molecule transporters with individual genes predominantly being ABC transporters.

**FIGURE 8:**
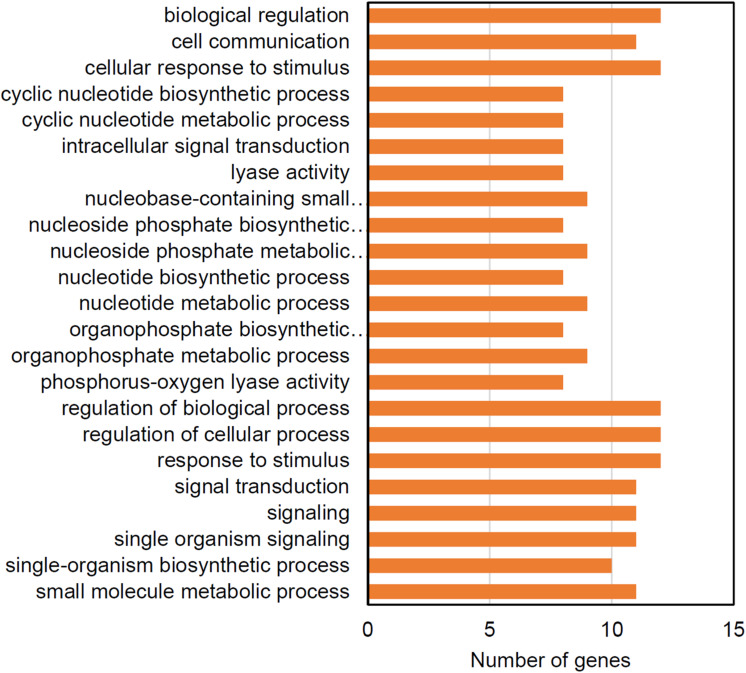
Enriched GO terms for genes showing evidence of purifying selection in the WT strain.

The WT strain also had significant enrichments for genes under purifying selection (Figure 8). More than half of these genes are predicted to function in regulating the levels of cyclic nucleotide second messengers suggesting that second messenger signal transduction may be under selective pressure in in the culture bag growth system. The *cw15* strain had no significantly-enriched GO terms for genes under purifying selection.

## Discussion

This EVT validated a strategy for identifying genes required by Chlamydomonas during log phase growth in spaceflight. We have shown that commercial FEP tissue culture bags can be used for batch culture of microalgae and result in 9–11 cell doublings during a 6 day culture. Cells in these cultures that divide normally will be at least 1000 fold enriched at stationary phase over cells with genetic variants that prevent mitotic division. Competition through multiple passages will eliminate mutations that prevent mitotic growth. Chlamydomonas is viable in these bags after prolonged dark storage, which enables full genome sequencing and identification of mutant genes in the culture. We have since used this strategy to grow the WT and *cw15* strains during the SpaceX CRS-15 mission; analysis of the spaceflight experiment is on-going.

Whole genome sequencing has been used to assess the mutagenic load of the bacteria, *Staphylococcus aureus*, in a 2 week spaceflight exposure (Guo et al., 2015). Less than 40 SNPs were detected in the genome from spaceflight with similar numbers of SNPs detected on ground and in spaceflight. These data suggest that exogenous mutagenesis is necessary to gain adequate signal of selection in short-term competitive growth experiments.

A yeast selection experiment was completed in spaceflight by using a genome-wide deletion collection (Nislow et al., 2015). The competitive growth experiment measured the reduction in representation of bar-coded mutants over the course of ∼21 mitotic generations. This type of competitive growth identifies individual genes needed for growth. By contrast, UVC mutagenesis has a higher genetic load and generates a more diverse array of allele types to compete within the culture. Moreover, three serial batch cultures of Chlamydomonas are expected to give ∼30 mitotic generations potentially giving more signal of selection within the genome sequence data.

There are several limitations to the design of this competitive growth assay. The algae were grown in mixotrophic conditions with both acetate and light as sources of energy. The addition of acetate to the media promotes rapid growth of the cultures allowing up to four passages to be completed in a 1-month SpaceX commercial resupply mission. Consequently, genes under purifying selection are expected to include pathways to utilize acetate. Similarly, the Veggie growth chamber has a limited spectrum of lighting with three narrow wavelengths that do not include far-red light (Massa et al., 2016). Each wavelength has two to four light level settings, and the lighting needed to be enriched for red light to provide 80–100 μmol/m^2^/s of light to the culture bags. Limitations to random mutagenesis are that loss-of-function mutations in all genes are not represented in each biological replicate of the experiment and that multiple mutations are simultaneously selected in individual cells during mitotic divisions. Moreover, we found that all cultures had a high level of polymorphisms that were identical to previously described natural variants in laboratory strains (Gallaher et al., 2015). The sequencing depth and 30 mitotic generations of the experiment did not provide sufficient sensitivity to detect changes in allele frequencies of individual natural variants. Consequently, we focused on selection signatures in novel mutations that were unique to UV mutagenized cultures.

Ultraviolet light causes direct DNA damage to create cyclobutane pyrimidine dimers, and the UV mutation signature is typically biased toward C > T transitions (Ikehata and Ono, 2011). Chlamydomonas DNA readily forms pyrimidine dimers, and WT strains have a robust dark-repair pathway to repair 90–95% of the DNA damage directly within 24 h (Small, 1987). With whole genome sequencing, we observed enrichment for T > G and C > G transversions in the mutations resulting from the UV treatment instead of the expected C > T transitions. Incorporation of several oxidative products of guanine can promote C > G transversions in response to a variety of mutagens, including UV (Kino and Sugiyama, 2005). In human cancers, C > G base changes are associated with activation of AID/APOBEC cytidine deaminases followed by error-prone translesion synthesis (Forbes et al., 2017). Human cancers also have base substitution signatures that are enriched for T > G, such as COSMIC signatures 17b and 28 (Forbes et al., 2017). However, no specific mechanisms have been proposed for the specific enrichment of T > G transversions.

Intriguingly, we observed signatures of selection in DNA polymerase ζ, θ, and REV1 (Supplementary Table S1), which are associated with translesion synthesis (Sakamoto, 2019). In the human germ line, C > G mutations have been suggested to be caused by errors in double-stand break repair (Gao et al., 2019). DNA polymerase θ and DNA ligase 4 function in the non-homologous end joining double strand break repair pathway, and we found evidence of selection for both of these enzymes in the EVT (Pannunzio et al., 2018). Evidence for selection in double-strand break repair and translesion synthesis suggests that these pathways are likely relevant to the UV-induced mutations observed.

The low sequence coverage of this experiment creates risk in using the frequency of recovery for specific mutant alleles in determining whether specific genes are under positive or purifying selection. The number of alleles discovered is limited by the sequencing depth, and higher coverage is necessary to increase the power of the statistics to detect selection at a genome-wide level. Nevertheless, we were able to classify 476 genes in the WT strain and 503 genes in the *cw15* strain for purifying or positive selection based on mutations within coding sequences. Among these, 104 genes were enriched for molecular functions based on GO terms. In addition to DNA repair pathways, these analyses revealed signal transduction and other information processing functions including, chromatin reading, RNA processing, and translation to be enriched within selected genes. The enriched molecular functions are predicted to be processes necessary for Chlamydomonas to adapt to the UV mutagenesis, lack of media agitation, the diffusion of gases across the FEP membrane, and the lighting conditions in the Veggie unit.

Scalable production of Chlamydomonas in spaceflight has multiple potential applications. The species will accumulate lipids to about 20–25% of total biomass, which can be used as an organic chemical feedstock (Becker, 2007; Xu et al., 2018). Chlamydomonas also has high protein content of 40–60% of biomass making it a potential source of food. Although it is not yet designated as a GRAS organism by the FDA, Chlamydomonas is non-toxic; animal feeding studies show no harmful effects at 4 g algae biomass per kg body weight per day, the highest consumption levels tested (Murbach et al., 2018). Equivalent consumption for a 65 kg person would be approximately 260 g of algae powder per day.

As a genetic model organism, Chlamydomonas biomass composition can be further modified by mutagenesis or targeted gene editing. For example, starch over-accumulation mutants have been isolated that shift starch content from 15 to 30–35% of biomass (Koo et al., 2017). Likewise, CRISPR gene editing of the Chlamydomonas zeaxanthin epoxidase gene significantly increases carotenoids needed to prevent macular degeneration (Baek et al., 2018). The modified algae strain was used to supplement chicken feed to increase the zeaxanthin content of eggs. The myriad of potential applications for microalgae production in spaceflight justify direct investigation of the genes needed for liquid culture production. Our current study shows both feasibility for a spaceflight experiment and identifies a series of cellular information processing genes that are likely required for Chlamydomonas to adapt to batch culture in breathable plastic bags.

## Data Availability Statement

The whole-genome sequences generated for this study can be found in the GeneLab database https://genelab-data.ndc.nasa.gov/genelab/accession/GLDS-265/.

## Author Contributions

JZ, BR, KT, HH, FB, and AS developed and performed the batch culture and mutagenesis. JZ constructed whole genome sequencing libraries and preliminary processing of the data. BM, YH, MR, and AS completed genome sequence analysis and interpretation. AS, JZ, and BM wrote the manuscript. All authors reviewed and agree with the manuscript content.

## Conflict of Interest

The authors declare that the research was conducted in the absence of any commercial or financial relationships that could be construed as a potential conflict of interest.

## Footnotes

1 http://broadinstitute.github.io/picard/

## References

[B1] Ai W. Guo S. Qin L. Tang Y. (2008). Development of a ground-based space micro-algae photo-bioreactor. *Adv. Space Res.* 41 742–747. 10.1016/j.asr.2007.06.060

[B2] Baek K. Yu J. Jeong J. Sim S. J. Bae S. Jin E. (2018). Photoautotrophic production of macular pigment in a *Chlamydomonas reinhardtii* strain generated by using DNA-free CRISPR-Cas9 RNP-mediated mutagenesis. *Biotechnol. Bioeng.* 115 719–728. 10.1002/bit.26499 29150930

[B3] Bansal V. (2010). A statistical method for the detection of variants from next-generation resequencing of DNA pools. *Bioinformatics* 26 i318–i324. 10.1093/bioinformatics/btq214 20529923 PMC2881398

[B4] Bansal V. Tewhey R. Leproust E. M. Schork N. J. (2011). Efficient and cost effective population resequencing by pooling and in-solution hybridization. *PLoS One* 6:e18353. 10.1371/journal.pone.0018353 21479135 PMC3068187

[B5] Becker E. W. (2007). Micro-algae as a source of protein. *Biotechnol. Adv.* 25 207–210. 10.1016/j.biotechadv.2006.11.002 17196357

[B6] Bolger A. M. Lohse M. Usadel B. (2014). Trimmomatic: a flexible trimmer for Illumina sequence data. *Bioinformatics* 30 2114–2120. 10.1093/bioinformatics/btu170 24695404 PMC4103590

[B7] Brechignac F. Schiller P. (1992). Pilot CELSS based on a maltose-excreting *Chlorella*: concept and overview on the technological developments. *Adv. Space Res.* 12 33–36. 10.1016/0273-1177(92)90006-j 11537074

[B8] Bretschneider J. Belz S. Helisch H. Detrell G. Keppler J. Fasoulas S. (2016). “Functionality and setup of the algae based ISS experiment,” in *Proceedings of the 46th International Conference on Environmental Systems* (Vienna: International Conference on Environmental Systems, Inc).

[B9] Buono S. Langellotti A. L. Martello A. Rinna F. Fogliano V. (2014). Functional ingredients from microalgae. *Food Funct.* 5 1669–1685. 10.1039/c4fo00125g 24957182

[B10] Caporgno M. P. Mathys A. (2018). Trends in microalgae incorporation into innovative food products with potential health benefits. *Front. Nutr.* 5:58. 10.3389/fnut.2018.00058 30109233 PMC6080594

[B11] Cingolani P. Platts A. Wang L. L. Coon M. Nguyen T. Wang L. (2012). A program for annotating and predicting the effects of single nucleotide polymorphisms, SnpEff: SNPs in the genome of *Drosophila melanogaster* strain w1118; iso-2; iso-3. *Fly* 6 80–92. 10.4161/fly.19695 22728672 PMC3679285

[B12] DePristo M. A. Banks E. Poplin R. Garimella K. V. Maguire J. R. Hartl C. (2011). A framework for variation discovery and genotyping using next-generation DNA sequencing data. *Nat. Genet.* 43 491–498. 10.1038/ng.806 21478889 PMC3083463

[B13] Forbes S. A. Beare D. Boutselakis H. Bamford S. Bindal N. Tate J. (2017). COSMIC: somatic cancer genetics at high-resolution. *Nucleic Acids Res.* 45 D777–D783. 10.1093/nar/gkw1121 27899578 PMC5210583

[B14] Gallaher S. D. Fitz-Gibbon S. T. Glaesener A. G. Pellegrini M. Merchant S. S. (2015). Chlamydomonas genome resource for laboratory strains reveals a mosaic of sequence variation, identifies true strain histories, and enables strain-specific studies. *Plant Cell* 27 2335–2352. 10.1105/tpc.15.00508 26307380 PMC4815092

[B15] Gao Z. Moorjani P. Sasani T. A. Pedersen B. S. Quinlan A. R. Jorde L. B. (2019). Overlooked roles of DNA damage and maternal age in generating human germline mutations. *Proc. Natl. Acad. Sci. U.S.A* 116 9491–9500. 10.1073/pnas.1901259116 31019089 PMC6511033

[B16] Garrison E. Marth G. (2012). Haplotype-based variant detection from short-read sequencing. *arXiv.org*[[Preprint].

[B17] Giardi M. Rea G. Lambreva M. Antonacci A. Pastorelli S. Bertalan I. (2013). Mutations of Photosystem II D1 protein that empower efficient phenotypes of *Chlamydomonas reinhardtii* under extreme environment in space. *PLoS One* 8:e64352. 10.1371/journal.pone.0064352 23691201 PMC3653854

[B18] Gorman D. S. Levine R. P. (1965). Cytochrome f and plastocyanin: their sequence in the photosynthetic electron transport chain of *Chlamydomonas reinhardi*. *Proc. Natl. Acad. Sci. U.S.A.* 54 1665–1669. 10.1073/pnas.54.6.1665 4379719 PMC300531

[B19] Guo J. Han N. Zhang Y. Wang H. Zhang X. Su L. (2015). Use of genome sequencing to assess nucleotide structure variation of *Staphylococcus aureus* strains cultured in spaceflight on Shenzhou-X, under simulated microgravity and on the ground. *Microbiol. Res.* 170 61–68. 10.1016/j.micres.2014.09.001 25304992

[B20] Helisch H. Keppler J. Detrell G. Belz S. Ewald R. Fasoulas S. (2019). High density long-term cultivation of *Chlorella vulgaris* SAG 211-12 in a novel microgravity-capable membrane raceway photobioreactor for future bioregenerative life support in space. *Life Sci. Space Res.* 24 91–107. 10.1016/j.lssr.2019.08.001 31987484

[B21] Ikehata H. Ono T. (2011). The mechanisms of UV mutagenesis. *J. Radiat. Res.* 52 115–125. 10.1269/jrr.10175 21436607

[B22] Katagiri M. Satoh A. Tsuji S. Shirasawa T. (2012). Effects of astaxanthin-rich *Haematococcus pluvialis* extract on cognitive function: a randomised, double-blind, placebo-controlled study. *J. Clin. Biochem. Nutr.* 51 102–107. 10.3164/jcbn.D-11-00017 22962526 PMC3432818

[B23] Kindle K. L. Schnell R. A. Fernández E. Lefebvre P. A. (1989). Stable nuclear transformation of *Chlamydomonas* using the *Chlamydomonas* gene for nitrate reductase. *J. Cell Biol.* 109 2589–2601. 10.1083/jcb.109.6.2589 2592399 PMC2115893

[B24] Kino K. Sugiyama H. (2005). UVR-induced G-C to C-G transversions from oxidative DNA damage. *Mutat. Res.* 571 33–42. 10.1016/j.mrfmmm.2004.10.010 15748636

[B25] Kliphuis A. M. Klok A. J. Martens D. E. Lamers P. P. Janssen M. Wijffels R. H. (2012). Metabolic modeling of *Chlamydomonas reinhardtii*: energy requirements for photoautotrophic growth and maintenance. *J. Appl. Phycol.* 24 253–266. 10.1007/s10811-011-9674-3 22427720 PMC3289792

[B26] Koo K. M. Jung S. Lee B. S. Kim J. B. Jo Y. D. Choi H. I. (2017). The mechanism of starch over-accumulation in *Chlamydomonas reinhardtii* high-starch mutants identified by comparative transcriptome analysis. *Front. Microbiol.* 8:858. 10.3389/fmicb.2017.00858 28588557 PMC5440458

[B27] Li H. (2013). Aligning sequence reads, clone sequences and assembly contigs with BWA-MEM. *arXiv.org* [Preprint].

[B28] Liu X. Luo Q. Cao Y. Goulette T. Xiao H. (2016). Mechanism of different stereoisomeric astaxanthin in resistance to oxidative stress in caenorhabditis elegans. *J. Food Sci.* 81 H2280–H2287. 10.1111/1750-3841.13417 27527357

[B29] Massa G. D. Wheeler R. M. Morrow R. C. Levine H. G. (2016). “Growth chambers on the International space station for large plants,” in *Proceedings of the VIII International Symposium on Light in Horticulture*, Vol. 1134 eds Currey C. J. Lopez R. G. Runkle E. S. (Belgium: ISHS Acta Horticulturae), 215–222. 10.17660/ActaHortic.2016.1134.29

[B30] Matula E. E. Nabity J. A. (2019). Failure modes, causes, and effects of algal photobioreactors used to control a spacecraft environment. *Life Sci. Space Res.* 20 35–52. 10.1016/j.lssr.2018.12.001 30797433

[B31] McKenna A. Hanna M. Banks E. Sivachenko A. Cibulskis K. Kernytsky A. (2010). The genome analysis toolkit: a mapreduce framework for analyzing next-generation DNA sequencing data. *Genome Res.* 20 1297–1303. 10.1101/gr.107524.110 20644199 PMC2928508

[B32] Merchant S. S. Prochnik S. E. Vallon O. Harris E. H. Karpowicz S. J. Witman G. B. (2007). The *Chlamydomonas* genome reveals the evolution of key animal and plant functions. *Science* 318 245–250. 10.1126/science.1143609 17932292 PMC2875087

[B33] Mergenhagen D. Mergenhagen E. (1989). The expression of a circadian rhythm in two strains of *Chlamydomonas reinhardii* in space. *Adv. Space Res.* 9 261–270. 10.1016/0273-1177(89)90082-3 11537341

[B34] Murbach T. S. Glávits R. Endres J. R. Hirka G. Vértesi A. Béres E. (2018). A toxicological evaluation of *Chlamydomonas reinhardtii*, a green Algae. *Int. J. Toxicol.* 37 53–62. 10.1177/1091581817746109 29303016

[B35] Nelson C. W. Moncla L. H. Hughes A. L. (2015). SNPGenie: estimating evolutionary parameters to detect natural selection using pooled next-generation sequencing data. *Bioinformatics* 31 3709–3711. 10.1093/bioinformatics/btv449 26227143 PMC4757956

[B36] Newman S. M. Boynton J. E. Gillham N. W. Randolph-Anderson B. L. Johnson A. M. Harris E. H. (1990). Transformation of chloroplast ribosomal RNA genes in *Chlamydomonas*: molecular and genetic characterization of integration events. *Genetics* 126 875–888. 1981764 10.1093/genetics/126.4.875 PMC1204285

[B37] Niederwieser T. Kociolek P. Klaus D. (2018). Spacecraft cabin environment effects on the growth and behavior of *Chlorella vulgaris* for life support applications. *Life Sci. Space Res.* 16 8–17. 10.1016/j.lssr.2017.10.002 29475523

[B38] Nislow C. Lee A. Y. Allen P. L. Giaever G. Smith A. Gebbia M. (2015). Genes required for survival in microgravity revealed by genome-wide yeast deletion collections cultured during spaceflight. *Biomed. Res. Int.* 2015:976458. 10.1155/2015/976458 25667933 PMC4309212

[B39] Otsuka T. Shimazawa M. Inoue Y. Nakano Y. Ojino K. Izawa H. (2016). Astaxanthin protects against retinal damage: evidence from in vivo and in vitro retinal ischemia and reperfusion models. *Curr. Eye Res.* 41 1465–1472. 10.3109/02713683.2015.1127392 27158842

[B40] Pannunzio N. R. Watanabe G. Lieber M. R. (2018). Nonhomologous DNA end-joining for repair of DNA double-strand breaks. *J. Biol. Chem.* 293 10512–10523. 10.1074/jbc.TM117.000374 29247009 PMC6036208

[B41] Sakamoto A. N. (2019). Translesion synthesis in plants: ultraviolet resistance and beyond. *Front. Plant Sci.* 10:1208. 10.3389/fpls.2019.01208 31649692 PMC6794406

[B42] Salem N. Eggersdorfer M. (2015). Is the world supply of omega-3 fatty acids adequate for optimal human nutrition? *Curr. Opin. Clin. Nutr. Metab. Care* 18 147–154. 10.1097/MCO.0000000000000145 25635599

[B43] Shah M. M. Liang Y. Cheng J. J. Daroch M. (2016). Astaxanthin-producing green microalga *Haematococcus pluvialis*: from single cell to high value commercial products. *Front. Plant Sci.* 7:531. 10.3389/fpls.2016.00531 27200009 PMC4848535

[B44] Small G. (1987). Repair systems for nuclear and chloroplast DNA in *Chlamydomonas reinhardtii*. *Mutat. Res.* 181 31–35. 10.1016/0027-5107(87)90284-3

[B45] Tian T. Liu Y. Yan H. You Q. Yi X. Du Z. (2017). agriGO v2.0: a GO analysis toolkit for the agricultural community, 2017 update. *Nucleic Acids Res.* 45 W122–W129. 10.1093/nar/gkx382 28472432 PMC5793732

[B46] Wang G. Chen H. Li G. Chen L. Li D. Hu C. (2006). Population growth and physiological characteristics of microalgae in a miniaturized bioreactor during space flight. *Acta Astronaut.* 58 264–269. 10.1016/j.actaastro.2005.11.001

[B47] Wang X. Willen R. Wadstrom T. (2000). Astaxanthin-rich algal meal and vitamin C inhibit *Helicobacter pylori* infection in BALB/cA mice. *Antimicrob. Agents Chemother.* 44 2452–2457. 10.1128/AAC.44.9.2452-2457.2000 10952594 PMC90084

[B48] Wilm A. Aw P. P. Bertrand D. Yeo G. H. Ong S. H. Wong C. H. (2012). LoFreq: a sequence-quality aware, ultra-sensitive variant caller for uncovering cell-population heterogeneity from high-throughput sequencing datasets. *Nucleic Acids Res.* 40 11189–11201. 10.1093/nar/gks918 23066108 PMC3526318

[B49] Xu L. Cheng X. Wang Q. (2018). Enhanced lipid production in *Chlamydomonas reinhardtii* by Co-culturing with *Azotobacter chroococcum*. *Front. Plant Sci.* 9:741. 10.3389/fpls.2018.00741 30002662 PMC6032324

